# Optimal biopsy approach for detection of clinically significant prostate cancer

**DOI:** 10.1259/bjr.20210413

**Published:** 2021-08-06

**Authors:** Simona Ippoliti, Peter Fletcher, Luca Orecchia, Roberto Miano, Christof Kastner, Tristan Barrett

**Affiliations:** 1 Urology Department, The Queen Elizabeth Hospital NHS Foundation Trust, King’s Lynn, Norfolk, UK; 2 Urology Department, Cambridge University Hospitals, Cambridge, UK; 3 Urology Unit, Policlinico Tor Vergata Foundation, Rome, Italy; 4 Division of Urology, Department of Surgical Sciences, University of Rome Tor Vergata, Rome, Italy; 5 Radiology Department, Cambridge University Hospitals, Cambridge, UK

## Abstract

Prostate cancer (PCa) diagnostic and therapeutic work-up has evolved significantly in the last decade, with pre-biopsy multiparametric MRI now widely endorsed within international guidelines. There is potential to move away from the widespread use of systematic biopsy cores and towards an individualised risk-stratified approach. However, the evidence on the optimal biopsy approach remains heterogeneous, and the aim of this review is to highlight the most relevant features following a critical assessment of the literature. The commonest biopsy approaches are via the transperineal (TP) or transrectal (TR) routes. The former is considered more advantageous due to its negligible risk of post-procedural sepsis and reduced need for antimicrobial prophylaxis; the more recent development of local anaesthetic (LA) methods now makes this approach feasible in the clinic. Beyond this, several techniques are available, including cognitive registration, MRI–Ultrasound fusion imaging and direct MRI in-bore guided biopsy. Evidence shows that performing targeted biopsies reduces the number of cores required and can achieve acceptable rates of detection whilst helping to minimise complications and reducing pathologist workloads and costs to health-care facilities. Pre-biopsy MRI has revolutionised the diagnostic pathway for PCa, and optimising the biopsy process is now a focus. Combining MR imaging, TP biopsy and a more widespread use of LA in an outpatient setting seems a reasonable solution to balance health-care costs and benefits, however, local choices are likely to depend on the expertise and experience of clinicians and on the technology available.

## Introduction

Prostate cancer (PCa) is the commonest male cancer in the UK, with an estimated 12% increase between 2014 and 2035, translating to around 233/100,000 men by 2035.^1^ Despite this, a high proportion of tumours are considered indolent and do not require active treatment,^2^ making it important to adequately control cancer mortality as well as reducing overdiagnosis and overtreatment. Clinically significant prostate cancer (csPCa) is defined by the International Society of Urological Pathology (ISUP) as grade group ≥2 cancer (Gleason score ≥3 +4).^3^

Guidelines from the National Institute for Health and Care Excellence (NICE), the European Association of Urology (EAU) and the American College of Radiology (ACR) recommend pre-biopsy multiparametric magnetic resonance imaging (mpMRI) to localise suspicious lesions for subsequent targeting at biopsy, or to safely avoid in low risk cases.^4,5^ mpMRI incorporates high-resolution anatomical *T*_2_ weighted images (*T*_2_WI) and the functional sequences diffusion-weighted MRI (DWI) and dynamic contrast-enhanced (DCE) MRI, and should be performed and reported to the Prostate Imaging Reporting and Data System (PI-RADS) v. 2.1 standards.^6^ The costs of an MRI-led diagnostic service are estimated to be 14.6% higher than traditional TRUS biopsy pathways^7^ ; however, this assumes all males receive a biopsy procedure and avoiding this in a subset of males will likely overcome this differential, and may even lead to cost savings.^8^ Cost-effective analyses have suggested an mpMRI first approach, followed by TRUS MRI-targeted biopsies, is more cost-effective for detecting csPCa than a systematic TRUS biopsy first strategy.^9^

Once a decision to biopsy is made, how to biopsy then needs to be determined. There is potential to move away from the widespread use of systematic biopsy cores and towards an individualised risk-stratified approach. However, as the evidence on the optimal biopsy approach is still heterogeneous, the aim of this review is to highlight its most relevant features following a critical assessment of the literature.

### MRI as the initial diagnostic step

Pre-biopsy mpMRI can yield a 27–49% reduction of patients undergoing transrectal ultrasound (TRUS)-guided biopsy.^10–16^ A meta-analysis of seven robust trials containing 2582 pooled males found that MRI with or without a targeted biopsy offered a 57% increase in csPCa detection, a 33% decrease in the total number of biopsies, and a 77% reduction in cores per biopsy procedure with little to no benefit in adding systematic cores.^11^ The PRECISION trial further reported 13% fewer insignificant cancers in an MRI-targeted biopsy group compared with a systematic TRUS-biopsy group.^12^ However, it should be noted that MRI performance and outcomes are heavily dependent on the quality of the MR imaging sequences,^17,18^ patient-related factors,^19,20^ and the experience of the interpreting radiologist.^21,22^

In patients whose mpMRI is suggestive of csPCa, MRI-TB offers improved diagnostic sensitivity compared to TRUS-guided biopsy.^12,23^ However, pre-biopsy mpMRI in biopsy-naive patients may not completely avoid the need for systematic biopsy (SB), as tumour detection is consistently reported as being improved when systematic and targeted approaches are combined. Schoots et al^24^, suggest that MRI-targeted biopsies can be used in two different diagnostic pathways: the ‘combined pathway’, in which patients with a positive mpMRI undergo both systematic and targeted biopsy (TB) and patients with a prostate-specific antigen (PSA) density >0.15 ng ml^−1^/cc and a negative mpMRI undergo SB; and the “MRI pathway”, in which patients with a positive mpMRI undergo only MRI-TB, and patients with a negative mpMRI avoid biopsy.

### To biopsy or not?

A recent metanalysis showed MRI to have a negative predictive value (NPV) of 90.8% at a threshold of grade group ≥2,^25^ which improves to approximately 96–97% at a threshold of group ≥3.^10,25–27^ Furthermore, follow-up SB (within 3 years of negative MRI) shows patients have rate of development of PCa that is similar to the expected at 5%.^28^ Utilising MRI, with the possibility of integrating further variables to increase NPV, should increase clinicians’ confidence to avoid biopsies in MRI negative patients. Ultimately, decisions must be made on a case-by-case basis taking into account factors such as family history, co-morbidity and patients’ own approach to risk, however, where clinical suspicion is high, SB should still be considered.^28^

In the context of a previous negative biopsies but high risk of PCa, the decision to rebiopsy is typically guided by PSA (density >0.15 ng ml^−1^/cc or velocity >0.75 ng/ml/year),^29^ clinical findings and suspicion, initial MRI suspicion and possibly a repeat MRI. Further variables can again be incorporated to assess risk and augment the biopsy decision.

### Augmenting the biopsy decision-making process

The PSA density threshold of 0.15 ng ml^−1^/cc has been found to significantly increase the NPV (53%–95% for bpMRI scores of 1–2 and from 53 to 93% for bpMRI score of 3) and increase positive predict value (PPV) of MRI (7%–47% for a bpMRI score of 3 and from 47 to 74% for bpMRI scores of 4–5).^30^ These improvements to predictive values have been replicated in the repeat biopsy setting where utilising PSA density 0.2 ng ml^−1^/cc was found to give significant improvements to mpMRI predictive values (increased NPV in Likert 1–2 from 71 to 91%, increased PPV in Likert 3 from 9 to 44% and increased PPV in Likert 4–5 from 47 to 66%).^16^

The use of other predictive biomarkers such as the prostate health index density (PHID) has been trialled.^31^ This marker was found to have 92.3% sensitivity and 35.3% specificity for csPCa and the suggested cut-off 0.44 would have decreased unnecessary biopsies by 35.3% (at the cost of missing 7.7% csPCa).^32^ Additional genetic analysis, alongside clinical parameters and protein levels has been used in scoring tests such as Stockholm-3. This score combined with MRI significantly reduced the number of biopsies required, whilst also decreasing detection of GG1 PCa with non-inferiority in detecting of GG > 2 PCa in subsequent TB.^33^ Further variables which could be considered include urinary biomarkers, such as that used in SelectMDx, which have also been found to correlate with finding lesions at MRI and if combined with MRI improve predictions of biopsy outcome.^34^ Presently, there is no consensus on which of these biomarkers is most appropriate to combine into a risk stratified approach to prostate biopsy. Furthermore, using these variables as a qualifying step prior to MRI is currently not advised due to limited and discordant evidence. For instance, although the 4K score (total PSA, free PSA, intact PSA, and hK2) combined with MRI has been found to give improved detection of aggressive PCa, using this as a filter (7.5% cut-off) prior to MRI has been reported to miss 33% of aggressive PCa.^35^

Another way to augment the biopsy decision process is by considering other imaging technologies. Multiparametric ultrasound (mpUS) is a new imaging modality combining different ultrasound parameters including greyscale ultrasound, computerised images, Doppler and power Doppler techniques, contrast-enhanced ultrasound (CEUS), shear wave elastography and high-resolution microultrasound, achieving improved diagnostic performance in PCa.^36^ Promising results have been reported especially by using sonoelastography, contrast-enhanced ultrasound and high-resolution microultrasound, either alone or in combination.^37,38^ MpUS heralds the potential for an accurate imaging-based diagnostic approach accessible to the community at large, but formal large-scale validation and standardisation of mpUS against final pathology results are still lacking.^4^ Furthermore, this imaging modality is more invasive than MRI for patients and less accurate, for instance injection of CEUS microbubbles typically only allows for assessment of a small portion of the gland. However, rather than substituting MRI for lesion detection, mpUS may well prove to be a useful diagnostic tool to aid the biopsy process itself. Another potential use of mpUS might include guidance and monitoring the application of focal therapy,^39^ or performing follow-up imaging after treatment, but further research is advocated.

### Biopsy approach: transrectal (TR) route vs transperineal (TP) route

#### TR –infection and cost

TR biopsy can be performed quickly and under local anaesthetic (LA), and provides good access to posterior prostatic lesions, leading to high PCa detection rates.^40^ However, the approach incurs unavoidable contamination of the biopsy needle as it passes from the rectum into the prostate. This may be exacerbated by the presence of resistant Gram-negative bacteria (majority being *E. coli*) within the rectal flora.^41^ Contamination is reflected in the rates of infectious complications and sepsis post-TR biopsy, with 1.9% of patients requiring readmission to hospital and 10.4% needing medical assessment without readmission.^42^ Furthermore, a recent analysis has shown that the rates of significant sepsis post-TR biopsy have been increasing over time, with a rate of 0.4% observed in 2012–16 but 1.12% in 2017–2019.^43^ Following TR biopsy, the 28-day all-cause mortality post-TR biopsy in the UK is low at 0.07%, however, it is notable higher than for TP approaches (0.05%).^43^ Prophylactic antibiotic therapy is therefore standard practice for TR biopsy, but may further contribute to antibiotic resistance, particularly to fluoroquinolones.^44^ This may be partially countered by use of pre-procedural rectal swabs to identify resistant microbes (particularly ESBLs), with subsequent tailoring of antibiotic prophylaxis in relation to resistance.^45^

#### TP –lower infection and complication

TP biopsies avoid faecal contamination, leading to lower rates of post-procedural sepsis (0.42%) compared to TR (1.12%).^43^ Indeed, some studies suggest that rates of infectious complications post-TP biopsy are lower, even approaching 0%,^46,47^ thus requiring either no antibiotic prophylaxis^48^ or reduced antimicrobial usage with single-dose prophylaxis.^49,50^ The procedure is generally well tolerated,^51^ with the most frequent post-operative complication being the development of acute urinary retention,^49^ which positively correlates with the number of cores taken as well as prostate volume^52^ and may also reflect an increased number of transitional zone cores, being obtained with proximity to the urethra. In terms of cancer detection, TP is at least equal,^53^ if not superior to TR biopsy in detecting anterior tumours.^54^

Traditionally, TP biopsies have been performed under general anaesthetic (GA), using a template grid mounted on a stepper unit to perform a complete mapping of the prostate.^55^ TP GA template mapping biopsy miss less csPCa,^56,57^ but also overdiagnose low-risk disease^56^ and entail a higher cost, need for operating theatre time, anaesthetic support and associated risks.^58^ As a result, TP biopsy can take longer to schedule compared to TR,^15^ negatively impacting the UK government-led targets for diagnosing or excluding cancer in 50% of patients within 14 days and 95% within 28 days.^59^ To further mitigate this, local anaesthetic TP biopsies are being employed such as PrecisionPoint (Perineologic, Cumberland, MD)^60^ or CamPROBE,^61^ which can simplify the biopsy process and reduce the number of access points to one or two per side and can be performed within the setting of outpatient clinics. LA-TP biopsies offer equal cancer detection rates^62^ whilst achieving lower incidence of post-operative infection,^63^ and are well tolerated by patients^53,64^ although there is ongoing research into the best technique for delivering the anaesthetic.^65^ Statistical analysis also highlights the cost savings of clinic-based LA-TP approaches.^43^ Given these advantages, there has been recent momentum behind the movement to discontinue TR biopsies, a so-called “TREXIT”.^66^

### Scalability of TP

Classically, TP biopsies were introduced as a second-line investigation after primary TR biopsies had failed to ascertain presence of cancer, whilst clinical suspicion persisted. Based on this clinical need, core distribution protocols were devised with higher core numbers using a template grid, but more typically 18–24 cores.^55^ This technique delivers well-known, published oncological outcomes.^55,67,68^ Due to the multiple entry points and depth, LA was not feasible for the majority of patients, however, LA-TP approaches have been developed in recent years, which allow application in the office or outpatient settings.^48,61,69^ With or without fusion, these techniques deliver results at least equivalent to TR approaches and there is promising potential for techniques like the vector biopsies to be equivalent to fusion template-guided approaches.

### Biopsy method: cognitive targeting vs fused MRI/ultrasound (rigid/elastic) vs in-bore MRI

**Figure 1.**
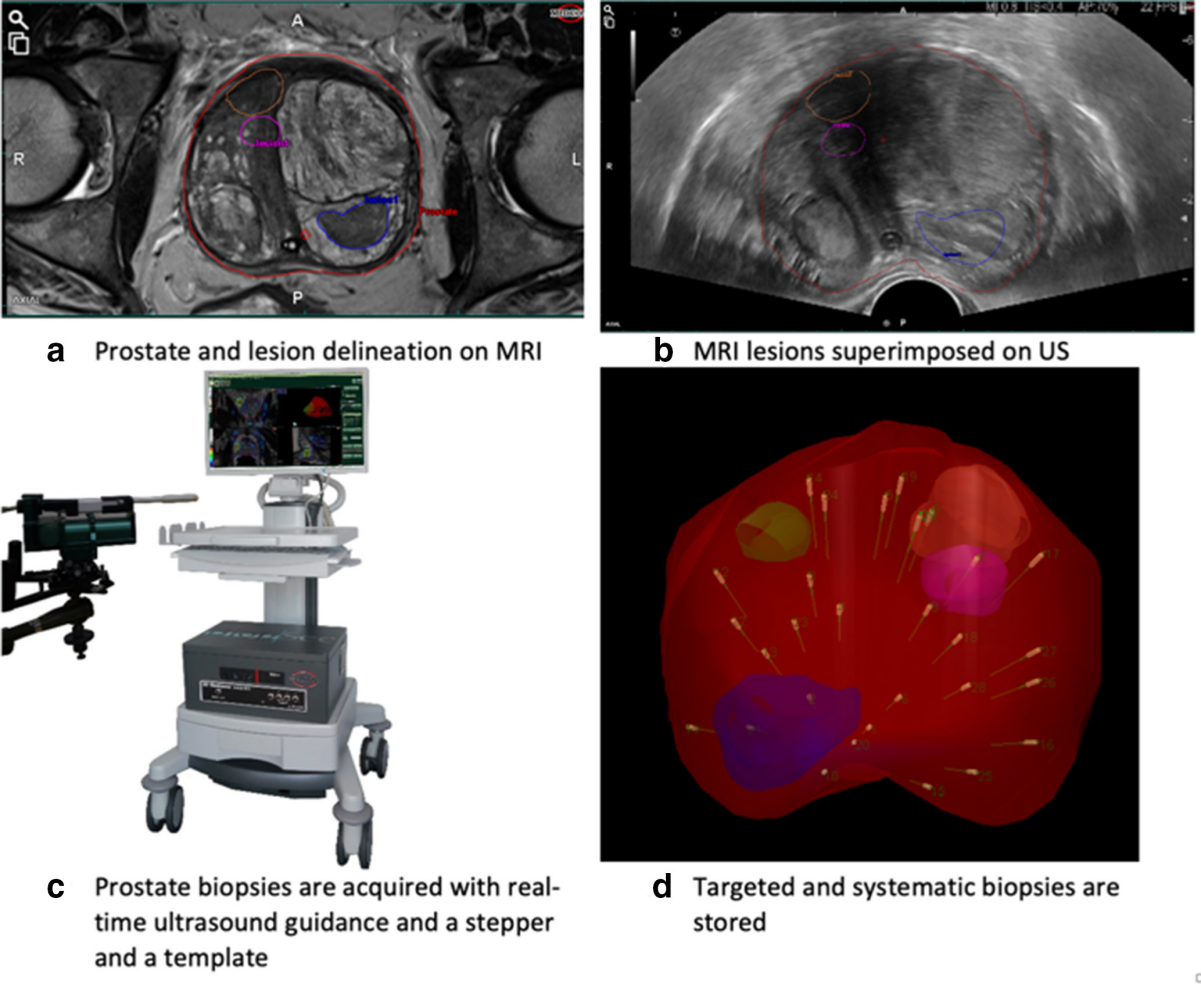
Example of an MRI fusion template-guided transperineal biopsy technique (BiopSeeTM, Medcom).

**Figure 2.**
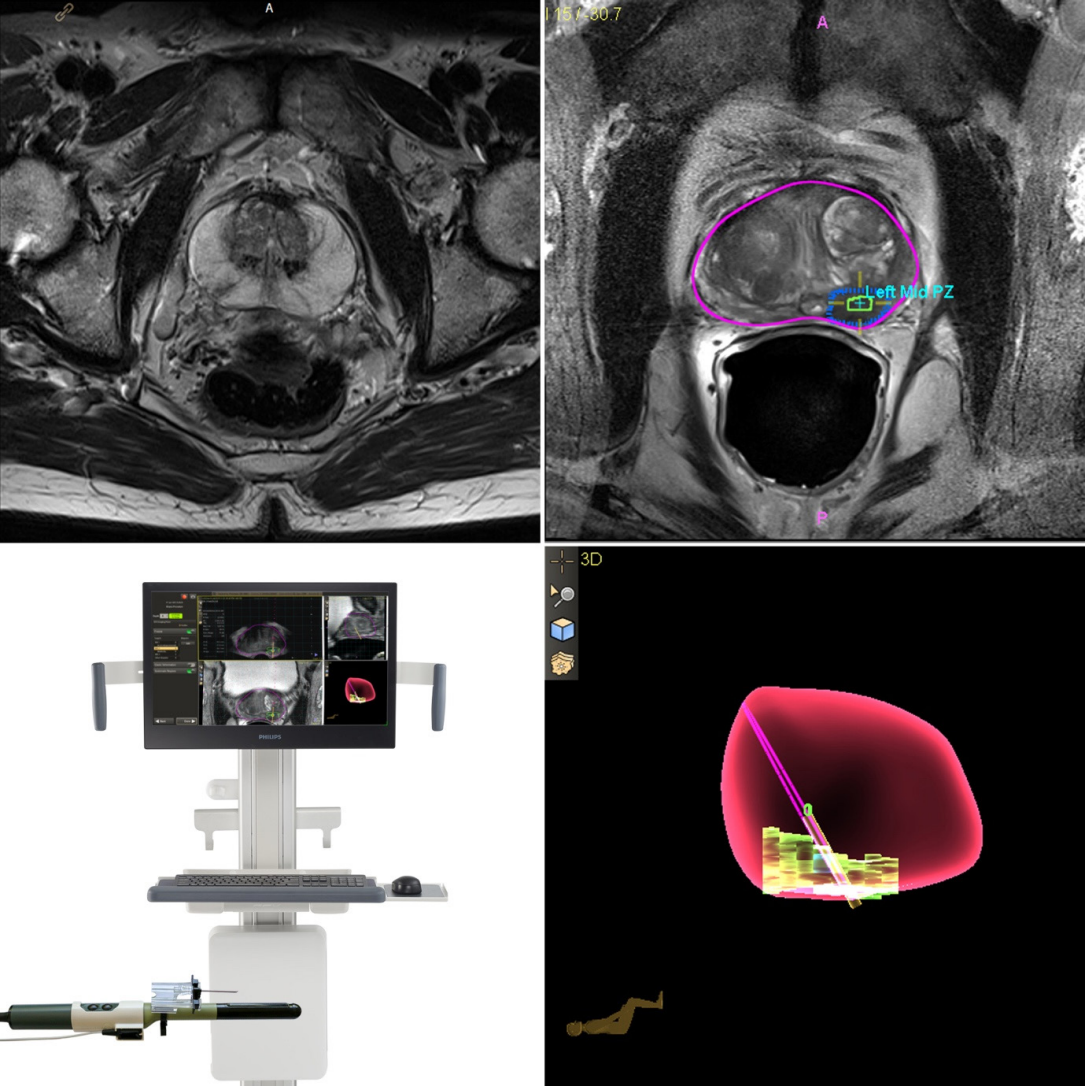
Example MRI fusion transperineal prostate biopsy using in-line needle guidance under local anaesthetic (UronavTM, Philips): Prostate MRI (top right) with contoured prostate (pink) and lesion (top left; green/blue); the needle guide is mounted to the probe (bottom left) to allow needle tracking within the sagittal plain; 3D animated documentation of the prostate, lesion and current plain.

The introduction of MRI-guided biopsies has changed the prostate biopsy paradigm. Existing strategies of MRI-guided biopsy techniques include direct MRI in-bore target biopsy which is performed in the MRI suite using real-time MRI guidance, MRI–ultrasound fusion in which MRI and TRUS images are fused using proprietary software (Figures 1 and 2), or visual estimation (otherwise known as cognitive registration) targeted biopsy in which the MRI is reviewed prior to biopsy by the operator and is used to cognitively target the MRI-identified lesion under TRUS guidance. All can be performed via either the transrectal or transperineal route. The FUTURE trial^70^ found no statistically significant difference in csPCa detection rates between TR cognitive, TP image fusion and TR in-bore targeting strategies. Similarly, the SmartTarget Biopsy^71^ and the PICTURE trials^72^ reported no significant difference in PCa detection rate between TP cognitive and MRI–ultrasound image fusion targeting techniques, although they suggested the combination of the two techniques may be better than each on its own, and the accuracy of the cognitive targeting approach is likely to be heavily experience dependent.^73^ Of note, TP cognitive targeting may be more reliable than TR when using a fixed grid, as the main targeting error arises only in the Z-plane, whereas for TR approaches, errors in any plane are possible.^74^ A multicentre cohort study showed that the image fusion technique may be superior in experienced hands.^75^ However, none of the studies provided data on whether differences may relate to variables such as prostate size, lesion characteristics, operator expertise and type of anaesthesia. A meta-analysis found that in-bore MRI-guided biopsy has improved overall PCa detection *vs* cognitive registration and MRI–ultrasound fusion biopsy.^23^ Furthermore, Costa et al proved in 2021 that MRI-guided in-bore biopsies had a lower incidence of grade group upgrades compared with MRI–ultrasound fusion biopsies, as another surrogate of sampling accuracy.^76^ However, in-bore biopsy takes significant magnet time and the equipment can be expensive, and does not allow for systematic cores to be obtained; assessment of the impact of these findings on patient outcomes and cost-utility analyses comparing the different techniques would be beneficial. Several MRI–ultrasound fusion biopsy platforms are commercially available and are summarised in Table 1. Rigid image fusion involves using landmarks to project the MRI prostate contour over the ultrasound image, whereas elastic fusion involves contouring the prostate on both MR and ultrasound images, with the fused contours then able to correct for prostate deformation and movement during the biopsy procedure. Although results are mixed in the literature, some studies suggest an accuracy advantage for elastic fusion over rigid fusion.^77,78^

**Table 1.** Commercially available MRI/ultrasound fusion systems

FUSION SYSTEM – TRADE NAME (MANUFACTURER)	Ultrasound IMAGE ACQUISITION	Ultrasound TRACKING MECHANISM	METHOD OF IMAGE REGISTRATION	BIOPSY ROUTE
Artemis (Eigen)	Manual rotation along a fixed axis (ultrasound probe on a tracking arm)	Mechanical arm with encoded joints	Elastic	TR
BioJet (Geoscan)	Real-time biplanar TRUS and 3D model of the prostate mounted on a positioning system	Stepper with 2-built-in encoders	Rigid	TP/TR
Biopsee (Pi Medical/MedCom)	Custom-made biplane TR US probe mounted on a stepper	Stepper with 2-built-in encoders	Rigid/Elastic	TP
HI RVS/Real-Time Virtual Sonography (Hitachi)	Real-time biplanar TRUS	Electromagnetic tracking	Rigid	TP/TR
UroNav (*In Vivo*/Philips)	Manual ultrasound 2D sweep. Freehand manipulation of ultrasound probe or mounted on a stepper	Electromagnetic tracking ultrasound	Rigid/Elastic	TR
Urostation (Koelis)	Automatic ultrasound probe rotation, three different volumes elastically registered	Image-based registration	Elastic	TR
Virtual Navigator (Esaote)	Manual ultrasound sweep. Freehand rotation of ultrasound probe	Electromagnetic tracking ultrasound and needle	Rigid	TR

TP, transperitoneal; TR, transrectal.

### Biopsy technique: target cores only vs saturation target approach vs systematic cores target + systematic cores

Several biopsy strategies have been proposed to investigate suspected PCa. For patients with non-suspicious MRI (Likert or PI-RADS <2) guidelines recommend considering omitting prostate biopsy, whilst those with suspicious MRI (Likert or PI-RADS >3) should be offered prostatic biopsy.

### How many cores?

In biopsy-naive patients with suspicious MRI lesions, the optimal biopsy technique in terms of number and type of cores to be taken is debated. The PRECISION, MRI-FIRST and 4M trials showed that TB (maximum four cores per target) in the PI-RADS 3–5 population gave superior detection rates of ISUP grade ≥2 and ≥3 cancers over a standard 12-core SB.^12,14^ Indeed, with a higher probability Likert 4–5 population other studies have found detection rates > 90% for TB alone^79^ whilst SB exclusively detect csPCa in only a small percentage of cases (1.9% of PI-RADS 4–5 or PI-RADS ≥3 with PSA density ≥0.12 ng ml^−1^).^80^ Furthermore, SB have been found to detect PCa with a higher Gleason grade than TB in only 3.2 and 5% of males with PI-RADS 4 and 5 MRI lesions respectively.^81^ The 4M trial did not find a significant difference in the detection of csPCa in TB *vs* SB, however, the results showed that TB detect fewer cases of insignificant PCa.^10^ This evidence suggests that TB are equal or even outperform SB in those with PI-RADS >3 lesions. However, there remains concern over TB missing or undergrading csPCa, particularly in the Likert/PI-RADS 3–4 population. Some studies in this group suggest up to 22% of csPCa could be missed by four core TB alone^82^ and perilesional biopsies (5 mm spaced around lesion perimeter) found higher grade group PCa than the TB in 8% of cases.^83^ Using a combined approach in PI-RADS 4–5 lesions was found to have a detection of Gleason 7–10 cancer of 71%, superior to the 59% for TB only and 61% for SB only. For PI-RADS 3 lesions, there was again superiority of a combined approach: 30 *vs* 21% (TB) and 27% (SB).^68^ Combining TB + SB therefore has the advantage of increasing cancer detection rates, but at the cost of increasing core numbers. To overcome this, the approach of saturation target biopsy (STB) has been proposed in which two target cores, two cores in the target sector and two cores from the adjacent sectors are taken, and can achieve >90% detection of Gleason score >7 PCa.^67^ When compared to extended prostatic biopsy a recent meta-analysis of 11,997 patients undergoing TRUS-guided prostate biopsy showed that STB had a significant advantage in biopsy-naive males, particularly those with PSA <10 ng ml^−1^, prostate volume >40 cc or PSA density <0.25 ng ml^−1^/cc.^57^

In the setting of previously negative biopsy, but high risk for csPCa, TB should be combined with SB or a Saturation-TB approach should be performed. One study found that SB + TB detected csPCa in 17.2% of cases, but in 60.7% of these csPCa was found in the systematic cores alone with only 28.5% being present in the target cores alone.^84^ Furthermore, in a small (*n* = 25) PI-RADS 3 population undergoing repeat biopsy, TB alone failed to detect 56% of csPCa, whereas SB only missed 4%.^85^

### Risk stratified approach

The variation in possible approaches has led to several viable alternatives to MRI-directed diagnostic strategies. Schoots et al^24^ proposed possible pathways for MRI directed diagnostic work-up. Their detection focused pathway maximises diagnostic yield by performing TB + SB or SB alone if MRI negative in all suspected PCa cases. The cost of this increased yield will be increased biopsies, core numbers and overdiagnosis of insignificant PCa, Conversely, a triage focussed pathway which only utilises TB only in those with positive MRIs will reduce detection of insignificant PCa, but will also reduce the detection of csPCa as patients with negative MRIs will not undergo biopsy and TB alone will inevitably underdiagnose/undergrade some csPCa. The latter is avoided in an “MRI-focused“ pathway in which MRI positive males will undergo TB and SB and STB may also be considered in this approach. MRI-based pathways could then be supplemented with further variables, to form an individualised “risk-stratified” pathway.

### Other management scenarios

#### Active surveillance

The increasing role of MRI in active surveillance reduces the need for biopsy in follow-up,^86^ and may also permit TB cores alone, which is appealing as the majority of PCa progression occur at the site of lesions previously demonstrated on MRI.^87^ The reduced post-operative complications associated with fewer biopsy cores is particularly beneficial in this population which is, by definition, considered to be of “low risk“. Although STB in active surveillance have been found to achieve 19.5% detection, with a significantly higher positive rate (57%) in those with smaller prostates (volume <37 cm^3^),^88^ other studies have found that standard SB and STB provided no additional benefit in detection of csPCa.^89^

#### Focal therapy (FT) work-up

Whole-gland removal or irradiation is considered the gold-standard for curative oncological treatment for localised PCa.^90^ However, it is often associated with sexual and urinary impairment that adversely affects patients’ quality of life.^91^ This has led to increased interest in developing ablative focal therapies for the treatment of localised, low to intermediate-risk PCa to minimise morbidity, provided that effective cancer control is ensured.^92^ A consensus group reported that tumour foci less than 1.5 ml on mpMRI or less than 20% of the prostate are suitable for FT, or up to 3 ml or 25% if localised to one hemi-gland.^92^ In the presence of an mpMRI-suspicious lesion, histological confirmation was deemed necessary and systematic biopsy remains essential to assess mpMRI-negative areas^92^ ; however, adequate criteria for systematic biopsy remains unresolved. The current gold-standard for characterising males who are considering FT is TP biopsy using a template-guided approach.^93,94^ When used with a 5 mm sampling frame, this approach can rule-in and rule-out PCa foci of 0.5 cc and 0.2 cc volume with 90% certainty.^95^ For patients who have not had an mpMRI, it was agreed that only a full TP template–mapping biopsy was sufficient to perform FT.^96^ As FT represents an emerging field, there is still a lack of high-quality evidence and prospective clinical trials and multicentre studies need to be prioritised to provide more robust guidance.^97^

### Future developments: bpMRI vs mpMRI-guided biopsy

Pre-biopsy biparametric MRI (bpMRI), which only uses *T*_2_WI and DWI, has also been considered in the general population in order to improve MRI accessibility, reduce costs and avoid potential immediate and long-term adverse effects of paramagnetic contrast medium administration.^98,99^ PRIME, an upcoming international multicentre prospective non-inferiority trial of bpMRI *vs* mpMRI for the diagnosis of csPCa, aims to provide high quality evidence on the role of prostate bpMRI.^100^

## Summary

**Figure 3.**
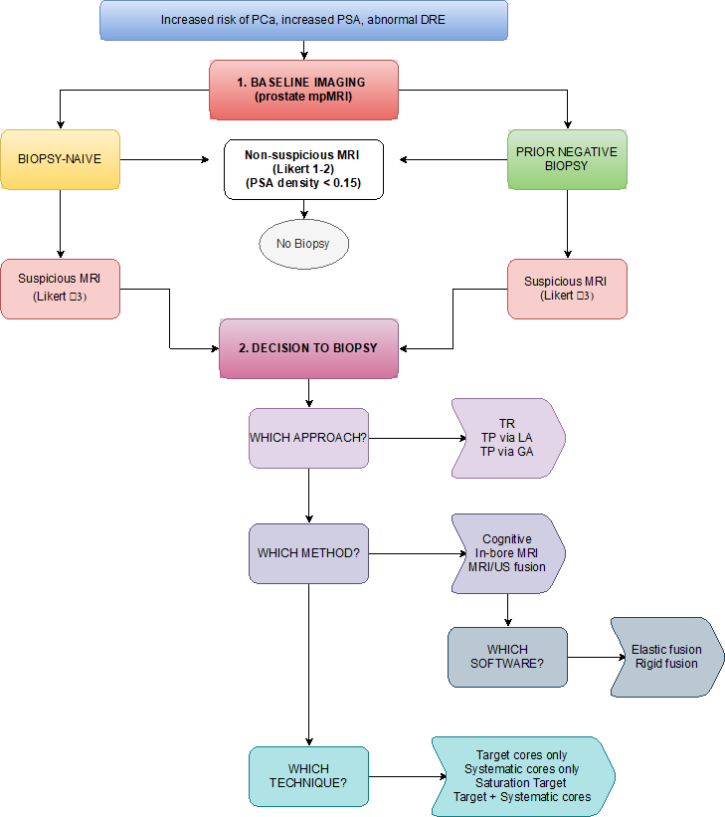
Flowchart of decision steps in prostate biopsy. SA, prostate-specific antigen; TR, transrectal; TP, transperitoneal.

The main features of current evidence in regards to prostatic biopsies are highlighted in Table 2. Stepwise decisional approach to biopsy is summarised in Figure 3 **.**

**Table 2.** Current evidence in prostate biopsy

	Evidence	References	Outcome
Biopsy Route			
TR	Higher and increasing rates of post-operative infection and sepsis, with associated antimicrobial concerns.	^41–45^	
TP via GA	Reduced post-operative infection whilst maintaining detection rates.	^43,46–54,66^	^*b*^, ^*c*^
TP via LA	Able to be performed in clinic and well tolerated by patients. Maintains equal detection.	^60–65^	^*b*^, ^*c*^
Biopsy method			
Cognitive	No superiority over in-bore MRI and MRI-TRUS fusion imaging biopsy methods. May be experience dependent	^70–72^	
In-bore MRI	Lower incidence of grade group upgrades and superior sampling accuracy compared to MRI-TRUS fusion biopsies. Superiority over cognitive registration and MRI-TRUS fusion imaging in overall PCa. No SB cores obtained	^23,76^	
MRI–TRUS fusion	Superior compared to cognitive biopsies if performed by experienced hands.	^75^	
Biopsy technique			
TB only	Reduced biopsy cores, associated with fewer complications. May risk undergrading cancer.	^10,12,14,79,81–83^	^*b*^, ^*c*^
SB only	More cores obtained, may be necessary if no target lesion or in work up for focal therapy.		
TB + SB	Increased detection and grading but high number of cores and associated increase in complications. Increased detected of insignificant PCa	^84^	^*a*^
STB	Supplements target biopsy to provide evaluation of surrounding zones giving increased detection and grading.	^67,88,89^	^*a*^, ^*b*^

GA, General anaesthesia; LA, Local anaesthesia; MRI, Magnetic resonance imaging; PCa, Prostate cancer; SB, Systematic biopsy; STB, Saturation target biopsy; TB, Target biopsy; TP, Transperineal; TR, Transrectal; TRUS, Transrectal ultrasound; csPCa, Clinically significant prostate cancer.

a Increased csPCa detection.

b Less side-effects.

c Less cost.

High level evidence shows that the widespread use of MRI has led to an improvement in csPCa detection, and a trend towards reduced number of cores per biopsy procedure with the pathway able to identify clinically significant disease and detect fewer insignificant cancers. Once the decision to biopsy is reached, how to biopsy then needs to be determined and the evidence here remains heterogenous. The TP route has significant advantages in terms of minimal post-operative infection and access to the anterior gland when compared to TR. However, general anaesthetic TP biopsies take longer to schedule compared to TR biopsy, impacting diagnostic target time. A move towards LA technique for TP biopsy and with fewer cores may help limit scheduling delays, further helping to meet proposed standards of the diagnostic timeframe. MRI/ultrasound fusion techniques represent a useful support for biopsy, especially when performed by an experienced operator. Despite this, the current literature shows no clear superiority in detection rates of MRI/ultrasound fusion over cognitive biopsy, however, reported studies have been from high-end centres with experienced operators, and further research in this field is advocated. MRI-guided in-bore biopsies is proved to be highly accurate, although high costs and limited availability of equipment may limit the generalisability of the method. Several biopsy techniques have been proposed, currently SB is recommended in the setting of a negative MRI and in the work-up of focal therapy, SB + TB is considered as the standard in patients with a MRI target, TB alone might be considered in the active surveillance setting, and saturation TB approaches have recently been proposed as a means of reducing the number of cores and biopsy-related complications, with high detection rates reported especially in patients with low PSA levels and small prostate volumes.

The MRI pathway in PCa diagnostics has evolved alongside the development of several biopsy methods and techniques. The currently available array of alternatives enables centres to offer biopsy procedures tailored to individual patient-specific risk, comorbidity and preference. Time to diagnosis and sustainability should be kept into account in the context of National Healthcare systems. Combining imaging, TP biopsy and a more widespread use of LA in an outpatient setting seems a reasonable solution to balance costs and benefits, however, local choices are likely to depend on the expertise and experience of clinicians and on the technology available.
